# Supplementary material for: Causal Relationship between Aging and Anorexia Nervosa: A White-Matter-Microstructure-Mediated Mendelian Randomization Analysis
Source: Biomedicines. 2024 Aug 16;12(8):1874. doi: 10.3390/biomedicines12081874 (PMC11351342; doi:10.3390/biomedicines12081874)
Supplement: Supplementary file 1 [file biomedicines-12-01874-s001.zip › Supplementary Figure/Figure S8/Leaveoneout plot of MR analysis for TL to ubm-b-1870.pdf]

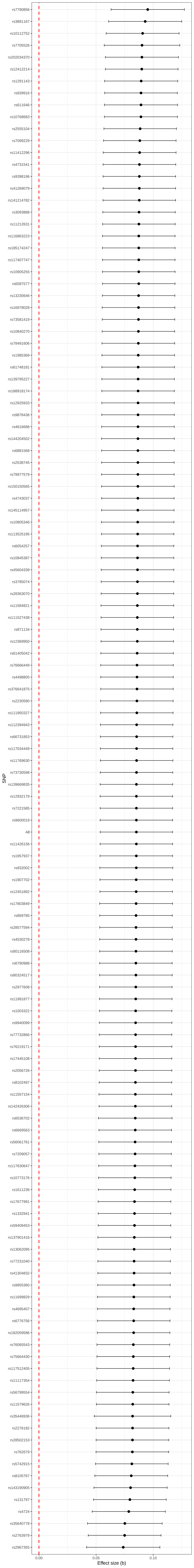
rs7790856
rs3891167
rs10112752
rs7705526
rs202034370
rs12412214
rs1291143
rs939916
rs611646
rs10768683
rs2555104
rs7099229
rs11412296
rs4731541
rs9398196
rs41269070
rs141214782
rs3093888
rs11212631
rs116863223
rs185174247
rs117407747
rs10905255
rs6587577
rs13230646
rs16978028
rs73581419
rs10840270
rs78491606
rs1985369
rs61748181
rs139795227
rs188918174
rs12925933
rs9878436
rs4616688
rs144204502
rs6881568
rs2538745
rs79977579
rs150150565
rs4743037
rs145114957
rs10805346
rs113525195
rs6054257
rs10845387
rs45604339
rs3785074
rs28363070
rs11584821
rs111527438
rs871134
rs12369950
rs61405042
rs76666449
rs4498805
rs376641875
rs2230590
rs111950327
rs112394943
rs66731853
rs117034449
rs11769630
rs73730598
rs139669835
rs12932179
rs7221585
rs9600019
A8
rs11426156
rs1957937
rs932002
rs1907702
rs12451892
rs17803849
rs869785
rs28577594
rs4530278
rs80116508
rs6790988
rs80324517
rs2977608
rs11991877
rs1003322
rs9940099
rs77732866
rs76219171
rs17445108
rs2056726
rs8102497
rs11557154
rs142426306
rs6536702
rs6669563
rs56061761
rs7209057
rs117630647
rs10773176
rs1611236
rs17677991
rs1332941
rs59409453
rs137901416
rs13062095
rs77231040
rs41304832
rs9955360
rs11699829
rs4695407
rs6776756
rs182059586
rs76065543
rs75664430
rs117512405
rs11117354
rs56799554
rs11579626
rs35446936
rs2276182
rs28502153
rs762679
rs5742915
rs8105767
rs143190905
rs131797
rs4724
rs35640778
rs2763979
rs2967355
SNP
0.00
0.05
0.10
Effect size (b)
